# Awareness, perceptions, and practices regarding transition to adult care for nephropathic cystinosis in Türkiye: a nationwide cross-sectional survey of pediatric nephrologists

**DOI:** 10.1186/s13023-026-04557-2

**Published:** 2026-08-26

**Authors:** Yeşim Özdemir Atikel, Sevcan A. Bakkaloğlu, Alev Yılmaz, Aslı Çelebi Tayfur, Aslı Kavaz Tufan, Aslıhan Kara, Ayşe Seda Pınarbaşı, Aysun Çaltık Yılmaz, Bahriye Atmış, Bedriye Nuray Alpman, Begüm Avcı, Burcu Yazıcıoğlu, Ece Demirci Bodur, Emre Leventoğlu, Erkin Serdaroğlu, Esra Nagehan Akyol Önder, Hülya Nalçacıoğlu, Kenan Yılmaz, Okan Akacı, Önder Yavaşcan, Özde Nisa Türkkan, Sare Gülfem Özlü, Seçil Arslansoyu Çamlar, Serra Sürmeli Döven, Songül Yılmaz, Yılmaz Tabel, Rezan Topaloğlu

**Affiliations:** 1 Department of Pediatric Nephrology, Eskisehir Health Practice and Research Center, University of Health Sciences, Eskisehir, Türkiye; 2 Department of Pediatric Nephrology, Eskisehir City Hospital, Eskisehir, Türkiye; 3 Department of Pediatric Nephrology, Gazi University Faculty of Medicine, Ankara, Türkiye; 4 Department of Pediatric Nephrology, Istanbul University Istanbul Faculty of Medicine, Istanbul, Türkiye; 5 Department of Pediatric Nephrology, Bolu Abant İzzet Baysal University Faculty of Medicine, Bolu, Türkiye; 6 Department of Pediatric Nephrology, Osmangazi University Faculty of Medicine, Eskisehir, Türkiye; 7 Department of Pediatric Nephrology, Firat University Faculty of Medicine, Elazig, Türkiye; 8 Department of Pediatric Nephrology, Yunus Emre State Hospital, Eskisehir, Türkiye; 9 Department of Pediatric Nephrology, University of Health Sciences, Etlik City Hospital, Ankara, Türkiye; 10 Department of Pediatric Nephrology, Cukurova University Faculty of Medicine, Adana, Türkiye; 11 Department of Pediatric Nephrology, Gülhane Faculty of Medicine, University of Health Sciences, Ankara, Türkiye; 12 Department of Pediatric Nephrology, Adana Başkent University Faculty of Medicine, Adana, Türkiye; 13 Dr Ece Demirci Bodur Pediatric Nephrology Clinic, Zonguldak, Türkiye; 14 Department of Pediatric Nephrology, Konya City Hospital, Konya, Türkiye; 15 Department of Pediatric Nephrology, Dr. Behçet Uz Children’s Hospital, İzmir, Türkiye; 16 Department of Pediatric Nephrology, Aksaray University Faculty of Medicine, Aksaray, Türkiye; 17 Department of Pediatric Nephrology, Ondokuz Mayıs University Faculty of Medicine, Samsun, Türkiye; 18 Department of Pediatric Nephrology, Sanliurfa Training and Research Hospital, Sanliurfa, Türkiye; 19 Department of Pediatric Nephrology, University of Health Sciences, Bursa Yuksek Ihtisas Training and Reseach Hospital, Bursa, Türkiye; 20 Department of Pediatric Nephrology, Medipol University Faculty of Medicine, Istanbul, Türkiye; 21 Department of Pediatric Nephrology, Marmara University Faculty of Medicine, Istanbul, Türkiye; 22 Department of Pediatric Nephrology, Ankara Yıldırım Beyazıt University, Faculty of Medicine, Ankara, Türkiye; 23 Department of Pediatric Nephrology, University of Health Sciences Izmir, Faculty of Medicine, Izmir, Türkiye; 24 Department of Pediatric Nephrology, Izmir City Hospital, Bayraklı, Izmir Türkiye; 25 Department of Pediatric Nephrology, Mersin University Faculty of Medicine, Mersin, Türkiye; 26 Department of Pediatric Nephrology, Ankara University Faculty of Medicine, Ankara, Türkiye; 27 Department of Pediatric Nephrology, İnönü University Faculty of Medicine, Malatya, Türkiye; 28 Department of Pediatric Nephrology, Hacettepe University Faculty of Medicine, Ankara, Türkiye

**Keywords:** Nephropathic cystinosis, Transition to adult care, Pediatric nephrology, Multidisciplinary care, Surveys and questionnaires, Health services research

## Abstract

**Background:**

Nephropathic cystinosis is a rare multisystem disorder in which transition from pediatric to adult care is particularly a vulnerable period because of lifelong treatment demands and the need for sustained multidisciplinary follow-up. We aimed to assess awareness, perceptions, and self-reported transition practices among pediatric nephrologists in Türkiye caring for patients with nephropathic cystinosis.

**Methods:**

We conducted a nationwide descriptive cross-sectional web-based survey of pediatric nephrologists in Türkiye who had longitudinally followed at least one patient with nephropathic cystinosis. A 59-item questionnaire covering awareness, perceptions, and self-reported practices was administered via Google Forms, and responses were analysed descriptively at the item level.

**Results:**

In this nationwide descriptive cross-sectional web-based survey, 48 pediatric nephrologists participated. Respondents worked mainly in university hospitals (47.9%) and training and research hospitals (39.6%) and had a median of 14 years of pediatric nephrology experience (IQR 6–17). Although 66.7% distinguished transition from transfer, familiarity with cystinosis-specific transition models was limited; only 16.7% had ever applied a structured model and 8.3% reported a written transition plan in their center. The most frequently perceived challenges were treatment non-adherence (89.6%) and disruption of multidisciplinary follow-up (87.5%). Most respondents reported marked differences between pediatric and adult services, and the median recommended age for initiating transition planning was 17.5 years (IQR 17–18). Overall, 91.7% supported the development of a national transition model (58.3% considered it definitely necessary and 33.3% necessary but difficult to implement without external support).

**Conclusions:**

Among pediatric nephrologists in Türkiye, awareness of transition-related risks in nephropathic cystinosis was high, but structured transition practices were uncommon and initiation of transition planning was generally recommended at a later age than suggested by current guidance. These findings indicate an implementation gap and support the development of a feasible national transition framework that can be adapted to local resources.

**Supplementary Information:**

The online version contains supplementary material available at https://doi.org/10.1186/s13023-026-04557-2.

## Introduction

Nephropathic cystinosis is a rare autosomal recessive lysosomal storage disorder caused by pathogenic variants in the *CTNS* gene that impair lysosomal cystine export and lead to progressive cystine accumulation across multiple tissues and organs [1, 2]. The classic infantile nephropathic form typically presents within the first year of life with renal Fanconi syndrome and, without cystine-depleting therapy, may progress to end-stage kidney disease (ESKD) during childhood [2–4]. Over the past few decades, cysteamine therapy and advances in kidney replacement therapy have improved survival [2, 4–8]. As a result, increasing numbers of patients with cystinosis now survive into adulthood and face a chronic multisystem disease with long-term extrarenal complications that require ongoing follow-up [2, 8–11].

In this context, transition from pediatric to adult care has become a vulnerable period that requires coordinated, continuous care [11–14], particularly given the persistent multisystem burden and complex long-term follow-up needs [12, 15, 16]. Here, “transition” should be distinguished from “transfer”: transition is a purposeful, planned process that supports adolescents and young adults in developing the skills and autonomy required for adult-oriented care, whereas transfer refers to the single handover event [12, 17]. Current nephrology guidance emphasizes early initiation, progressive readiness building, and structured information exchange between pediatric and adult teams [15, 17].

Cystinosis poses disease-specific challenges for transition, including lifelong therapy, adherence difficulties, and the need for sustained multidisciplinary coordination [12, 15, 17]. Cystinosis-focused recommendations and coordinated transition models have been proposed, incorporating elements such as standardized transfer documentation, defined roles, and joint pediatric-adult collaboration [12, 13, 18, 19]. However, implementation remains heterogeneous across health systems, and published data on routine transition practices in cystinosis are limited [11, 13, 15, 20]. To our knowledge, no transition protocol or readiness tool validated specifically for nephropathic cystinosis is currently available. Although expert-proposed cystinosis-specific transition models have been described internationally (e.g., the RISE protocol [13] and the T-CiS.bcn coordinated model [19]), none has undergone formal validation, and no national transition guideline or protocol has been established in Türkiye.

To date, no study in Türkiye has specifically examined clinicians’ awareness and routine practices regarding transition in cystinosis. Cystinosis-specific transition models have been proposed, but their real-world uptake is unknown and no national data describe current practice; because guidance cannot be uniform across rare diseases, disease- and context-specific evidence is needed. This study provides the first national baseline for cystinosis transition in Türkiye. We therefore conducted a national survey among pediatric nephrologists in Türkiye to assess awareness, perceptions, and self-reported transition practices, identify perceived barriers and risks, and inform future efforts toward locally adapted, cystinosis-specific transition approaches.

## Methods

### Study design and participants

We conducted a nationwide descriptive cross-sectional web-based survey of pediatric nephrologists in Türkiye. Eligible participants were pediatric nephrologists who had longitudinally followed at least one patient with nephropathic cystinosis at any point in their clinical practice. There were no restrictions based on geographic region, center size, center type, or current cystinosis patient load. The survey was deliberately focused on the pediatric (sending) side of transition as a first phase; assessment of the perspectives of adult nephrologists and of patients and families is planned for subsequent phases.

Because the study aimed to provide a descriptive national snapshot of awareness and practice, no formal sample size calculation was performed. Participants were recruited through voluntary non-probability sampling. The survey link was distributed through the official messaging group of the Turkish Pediatric Nephrology Association and three reminder messages were posted during the survey period. The exact number of pediatric nephrologists in Türkiye who were actively or previously involved in the longitudinal care of patients with cystinosis is not known; therefore, a definitive response rate could not be calculated.

### Questionnaire development

The questionnaire was developed by YOA after a focused review of the literature on (i) multidisciplinary management of cystinosis and (ii) healthcare transition principles in chronic kidney disease (CKD) and other lifelong conditions. Items were organized into five domains: (1) respondent and center characteristics; (2) conceptual understanding of “transition” versus “transfer”; (3) awareness of structured transition models, guidelines, and tools; (4) current practices related to preparation for transfer, multidisciplinary follow-up, collaboration with adult services, and post-transfer follow-up; and (5) perceived risks, barriers, and needs for improving transition.

The final questionnaire comprised 59 items, including single-choice questions, matrix items, and short-answer numeric items (e.g., years of professional experience and the number of specialists in the respondent’s center). The survey was administered in Turkish, and an English translation of the complete questionnaire is provided in Supplementary Material 1.

Before dissemination, the questionnaire was pretested by three pediatric nephrologists who were not involved in its development to assess clarity and face validity; no major concerns were raised. No formal psychometric validation study was performed. To support content coverage and face validity, items were derived from established transition concepts and cystinosis-specific recommendations and were pretested by clinical experts.

### Data collection and data management

The survey was administered using Google Forms (Google LLC, Mountain View, CA, USA). Participation was voluntary and uncompensated.

All questionnaire items were set as mandatory to prevent missing responses. To minimize duplicate entries, the Google Forms setting restricting responses to one per user was enabled. Although Google account sign-in may therefore have been required at the platform level, no account-related information was accessed, exported, or analyzed by the investigators, and no such information was linked to survey responses. The analytic dataset contained no direct identifiers.

The questionnaire did not request direct personal identifiers such as names, institutional identification numbers, or national identification numbers. Respondents who wished to be contacted for future co-design or consensus-based activities related to transition model or guidance development could optionally provide an email address in a separate opt-in field at the end of the survey. These contact details were stored separately from survey responses and were not linked to the analytic dataset.

### Ethical considerations

This study was approved by the Ethics Committee of Eskisehir City Hospital (approval no. ESH/BAEK2026/307; January 6, 2026). The requirement for written informed consent was waived by the Ethics Committee because this was a voluntary, non-interventional, web-based survey and the analytic dataset was deidentified. No direct personal identifiers were included in the analytic dataset. Completion of the questionnaire was considered implied consent for participation.

### Data analysis

A total of 48 unique completed questionnaires were included in the final dataset and formed the basis of the descriptive analyses. Categorical variables are summarized as counts (n) and percentages (%). Continuous variables are presented as median with interquartile range (IQR) or median with range (minimum-maximum), as appropriate. For matrix items, distributions across response categories were reported. No composite scores were calculated, and no formal hypothesis-testing analyses were performed. Because the survey was designed for item-level descriptive reporting and generated no composite scores or scales, internal-consistency reliability statistics (e.g., Cronbach’s alpha) were not applicable. As pre-specified exploratory analyses, key items were compared between respondents who rated themselves as very experienced and those who rated themselves as moderately or minimally experienced, and between those whose center currently followed an adult-aged patient with cystinosis and those who did not or were unsure, using Fisher exact tests. The number of cystinosis patients followed per clinician was not collected and could not be examined directly; these subgroup comparisons were exploratory and interpreted descriptively given the limited sample.

For the short-answer item asking about the perceived age or age range at which transition should be initiated, responses provided as ranges were summarized using the midpoint of the reported range for descriptive purposes and were additionally grouped into reporting categories.

The analyses were conducted using Microsoft Excel (Microsoft Corp., Redmond, WA, USA). Given the purely descriptive design and non-probability sample, no inferential statistical tests were applied.

## Results

### Characteristics of participating pediatric nephrologists and centers

A total of 48 pediatric nephrologists completed the survey. Respondent and center characteristics are summarized in Table 1. Most respondents worked in tertiary care settings, with 23 (47.9%) in university hospitals and 19 (39.6%) in training and research hospitals. The median duration of professional experience in pediatric nephrology was 14 years (IQR 6–17).

Pediatric nephrology teams were generally small, with a median of 3 specialists per center (range, 1–9). By comparison, adult nephrology teams within the same institutions were slightly larger, with a median of 4 specialists (range, 0–10); the lower bound reflects pediatric-only institutions where adult nephrology care is provided through external referral. Self-reported experience in managing cystinosis varied: 16 respondents (33.3%) described themselves as very experienced, 24 (50.0%) as moderately experienced, and 8 (16.7%) as having limited experience.

When asked whether adult patients with cystinosis were currently being followed up in their center, responses were distributed across three categories. Fifteen respondents (31.2%) reported ongoing follow-up in their center, an equal proportion reported no follow-up, and more than one-third (*n* = 18, 37.5%) indicated they were unsure.

**Table 1:** Institutional characteristics, professional experience and cystinosis-related experience of participating pediatric nephrologists (*n* = 48)

Characteristic	Value
**Type of institution**, n (%)	
University hospital	23 (47.9%)
Training and research hospital	19 (39.6%)
State hospital	3 (6.2%)
Private hospital	1 (2.1%)
Other	2 (4.2%)
**Professional experience in pediatric nephrology**, years	14 (6–17)*
**Pediatric nephrologists in current center**, n	3 (1–9)†
**Adult nephrologists in the same institution**, n	4 (0–10)†
**Self-rated experience in managing cystinosis**, n (%)	
Very experienced	16 (33.3%)
Moderately experienced	24 (50.0%)
Limited experience	8 (16.7%)
No experience	0 (0.0%)
**Adult-aged patients with cystinosis currently followed in the center**, n (%)	
Yes	15 (31.2%)
No	15 (31.2%)
Not sure	18 (37.5%)

Values are presented as n (%), median (IQR), or median (range), as indicated

*Median (interquartile range)

† Median (range, minimum–maximum)

### Multidisciplinary involvement in pediatric cystinosis care

The patterns of multidisciplinary follow-up varied considerably across specialties, as reported by the respondents (Table 2). Ophthalmology and metabolic medicine were the most consistently integrated disciplines: 37 of 48 respondents (77.1%) reported routine ophthalmology follow-up, while 26 respondents (54.2%) reported routine metabolic medicine follow-up. Endocrinology was also commonly involved, with 20 respondents (41.7%) reporting routine follow-up.

In contrast, several disciplines were predominantly engaged on a consultative basis. Most respondents selected “seen as needed” for neurology (81.2%), gastroenterology (83.3%), pulmonology (81.2%), urology (77.1%), physical therapy/rehabilitation (75.0%), psychiatry/psychology (85.4%) and psychosocial support services (72.9%).

The highest proportions of non-follow-up or unavailability were reported for supportive and rehabilitation-related services. One-fifth of the respondents reported that urology (20.8%) and physical therapy/rehabilitation (20.8%) were not routinely followed or not readily accessible, and 22.9% reported the same for psychosocial support services. Limited access was also noted for metabolic medicine (10.4%).

**Table 2:** Patterns of multidisciplinary follow-up for patients with cystinosis as reported by respondents (*n* = 48)

Discipline	Routinely follows, *n* (%)	Seen as needed, *n* (%)	Seen once yearly, *n* (%)	Not followed / unavailable, *n* (%)
Ophthalmology	37 (77.1%)	6 (12.5%)	4 (8.3%)	1 (2.1%)
Metabolic medicine	26 (54.2%)	17 (35.4%)	0 (0.0%)	5 (10.4%)
Endocrinology	20 (41.7%)	25 (52.1%)	1 (2.1%)	2 (4.2%)
Dietetics	8 (16.7%)	35 (72.9%)	1 (2.1%)	4 (8.3%)
Cardiology	7 (14.6%)	32 (66.7%)	7 (14.6%)	2 (4.2%)
Neurology	3 (6.2%)	39 (81.2%)	2 (4.2%)	4 (8.3%)
Gastroenterology	3 (6.2%)	40 (83.3%)	0 (0.0%)	5 (10.4%)
Psychosocial support services	2 (4.2%)	35 (72.9%)	0 (0.0%)	11 (22.9%)
Pulmonology	0 (0.0%)	39 (81.2%)	1 (2.1%)	8 (16.7%)
Urology	0 (0.0%)	37 (77.1%)	1 (2.1%)	10 (20.8%)
Physical therapy/rehabilitation	0 (0.0%)	36 (75.0%)	2 (4.2%)	10 (20.8%)
Psychiatry / Psychology	0 (0.0%)	41 (85.4%)	0 (0.0%)	7 (14.6%)

Values are presented as n (%)

### Awareness of transition terminology and structured transition concepts

As shown in Table 3, most pediatric nephrologists reported that “transition” and “transfer” are not synonymous: 32 of 48 (66.7%) perceived a clear distinction, whereas 7 (14.6%) reported no difference, and 9 (18.8%) were unsure. Awareness of the term “structured transition process” was evenly split: 24 respondents (50.0%) reported prior exposure, and 24 (50.0%) reported no prior exposure.

More than half of the respondents (*n* = 25, 52.1%) reported no knowledge of any cystinosis-specific transition model, 20 (41.7%) reported limited knowledge, and only 3 (6.2%) reported detailed knowledge. Eight respondents (16.7%) stated that they or their team had ever implemented a structured transition model for at least one patient with cystinosis.

**Table 3:** Awareness, familiarity, and implementation of structured transition concepts and cystinosis-specific models (*n* = 48)

Variable / response category	*n* (%)
**Perceives a difference between “transition” and “transfer”**	
Yes	32 (66.7%)
No	7 (14.6%)
Unsure	9 (18.8%)
**Has previously heard the term “structured transition process"**	
Yes	24 (50.0%)
No	24 (50.0%)
**Knowledge of any cystinosis-specific transition model / guideline / program**	
No knowledge	25 (52.1%)
Limited knowledge	20 (41.7%)
Detailed knowledge	3 (6.2%)
**Has ever applied a structured transition model for at least one cystinosis patient**	
Yes	8 (16.7%)
No	35 (72.9%)
Planning to implement soon	1 (2.1%)
Unsure / no opinion	4 (8.3%)

Values are presented as n (%)

### Perceived challenges during transition and system-level differences between pediatric and adult services

**Fig. 1:**
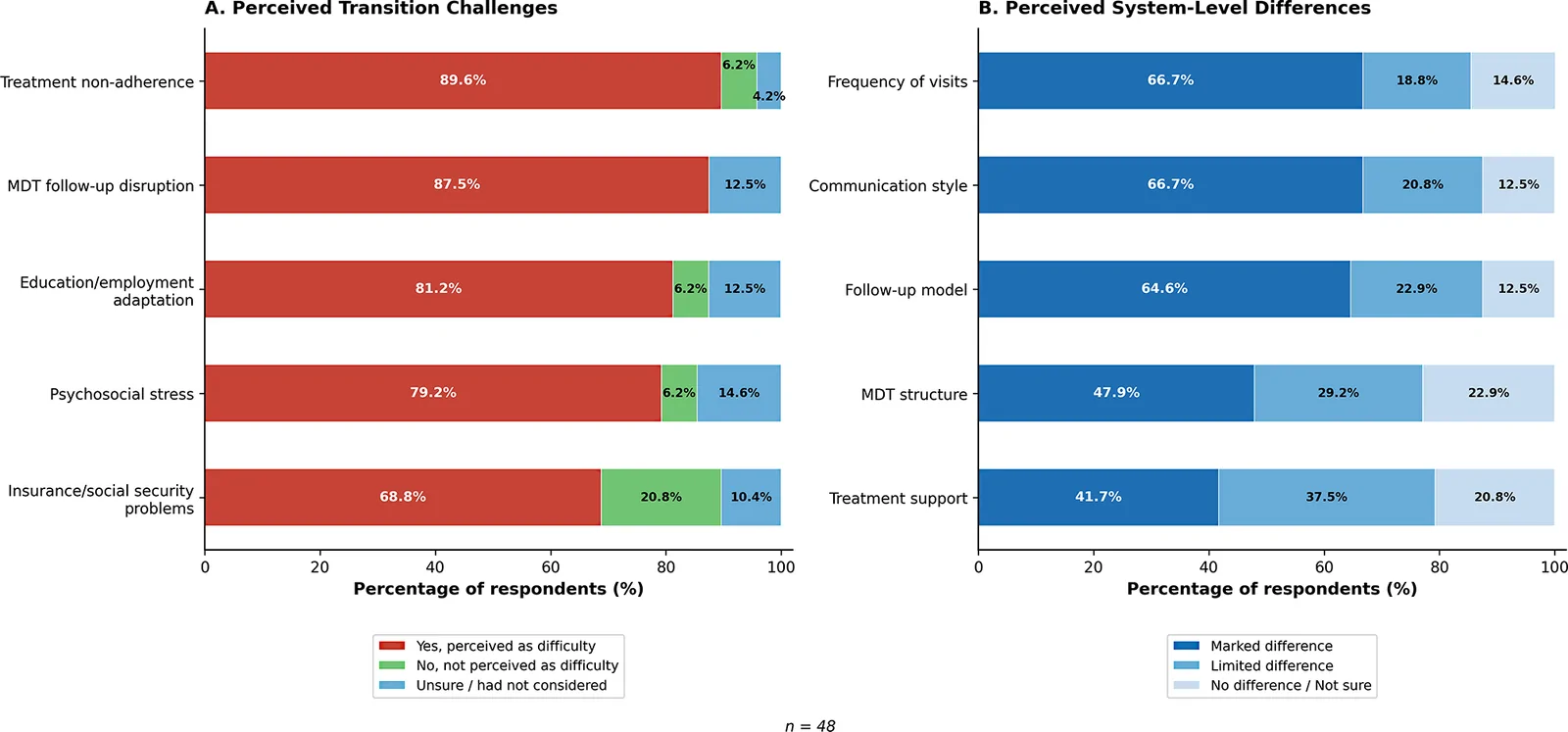
Perceived transition challenges (**A**) and system-level differences between pediatric and adult services (**B**) as reported by pediatric nephrologists (*n* = 48). (Responses reflect respondents’ reports about their own centers). MDT, multidisciplinary team

Respondents identified multiple challenges encountered by patients with cystinosis during the transition from pediatric to adult-oriented care (Fig. 1A). Disruption of multidisciplinary follow-up was reported as a difficulty by 42 respondents (87.5%), and treatment non-adherence (missed medication doses or appointments) by 43 (89.6%). Psychosocial stressors (e.g., identity, independence) were also perceived as a difficulty (*n* = 38, 79.2%). Social and structural challenges were also reported, including difficulties in adapting to education and employment (*n* = 39, 81.2%) and issues with social security/insurance-related difficulties (*n* = 33, 68.8%).

Overall, 45 respondents (93.8%) reported structural differences between pediatric and adult clinics, (Fig. 1B). Across the specific domains, the most frequently reported marked differences were in frequency of visits, (32 respondents, 66.7%), communication style, (32 respondents, 66.7%), and follow-up model, (31 respondents, 64.6%). Marked differences in multidisciplinary structures, (23 respondents, 47.9%) and treatment support, (20 respondents, 41.7%) were also reported.

### Current transition practices and organization of transfer

**Fig. 2:**
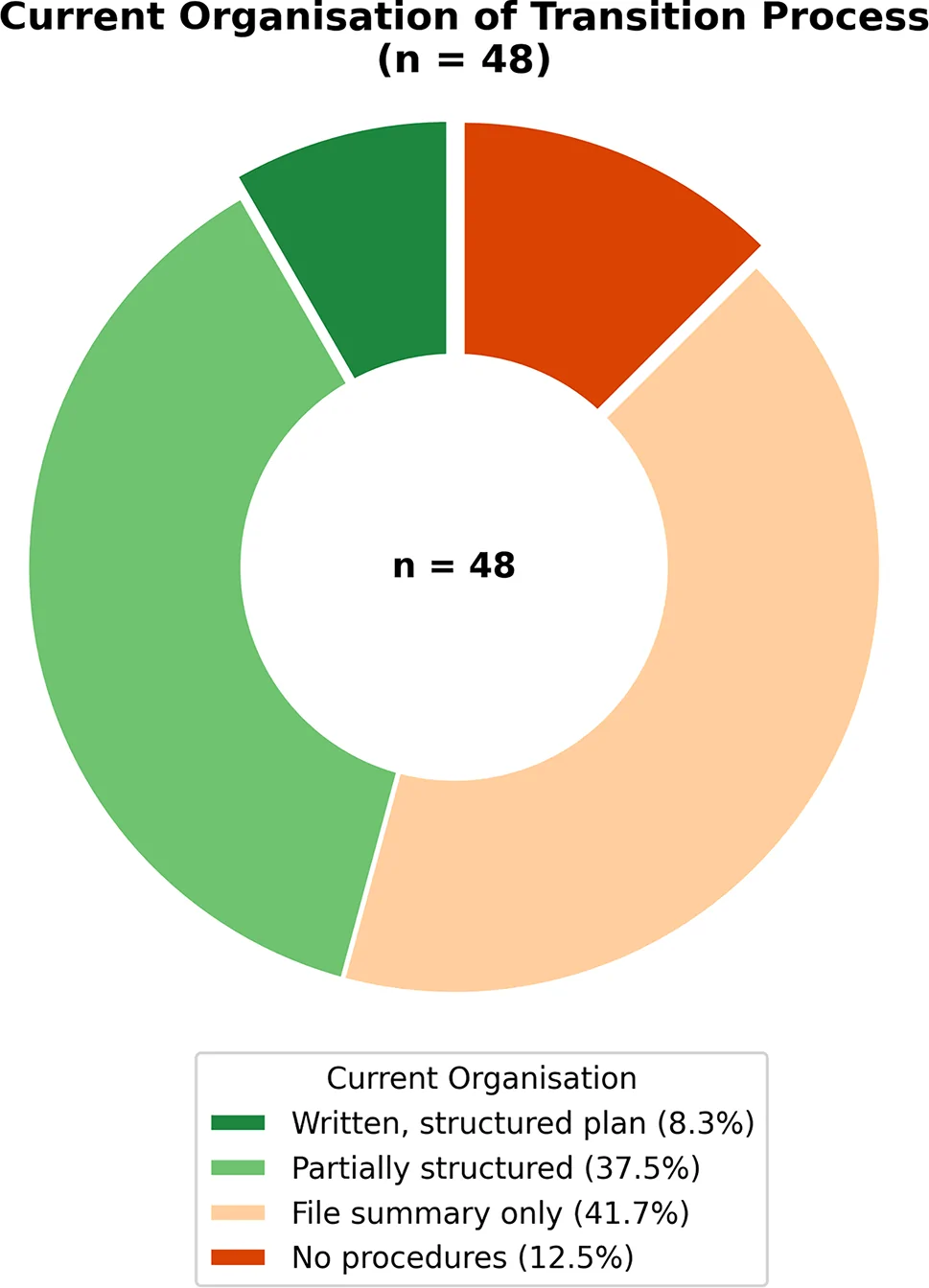
Current organization of the transition process for patients with cystinosis as reported by pediatric nephrologists (*n* = 48)

When respondents were asked how the transition process for patients with cystinosis was currently organized in their center, 4 of 48 (8.3%) reported having a written, structured transition plan. Most participants described either a partially structured process (*n* = 18, 37.5%) or a transfer based solely on a file summary (*n* = 20, 41.7%). Six respondents (12.5%) indicated that transition-specific procedures were not applied (Fig. 2).

### Perceived timing for starting transition

**Fig. 3:**
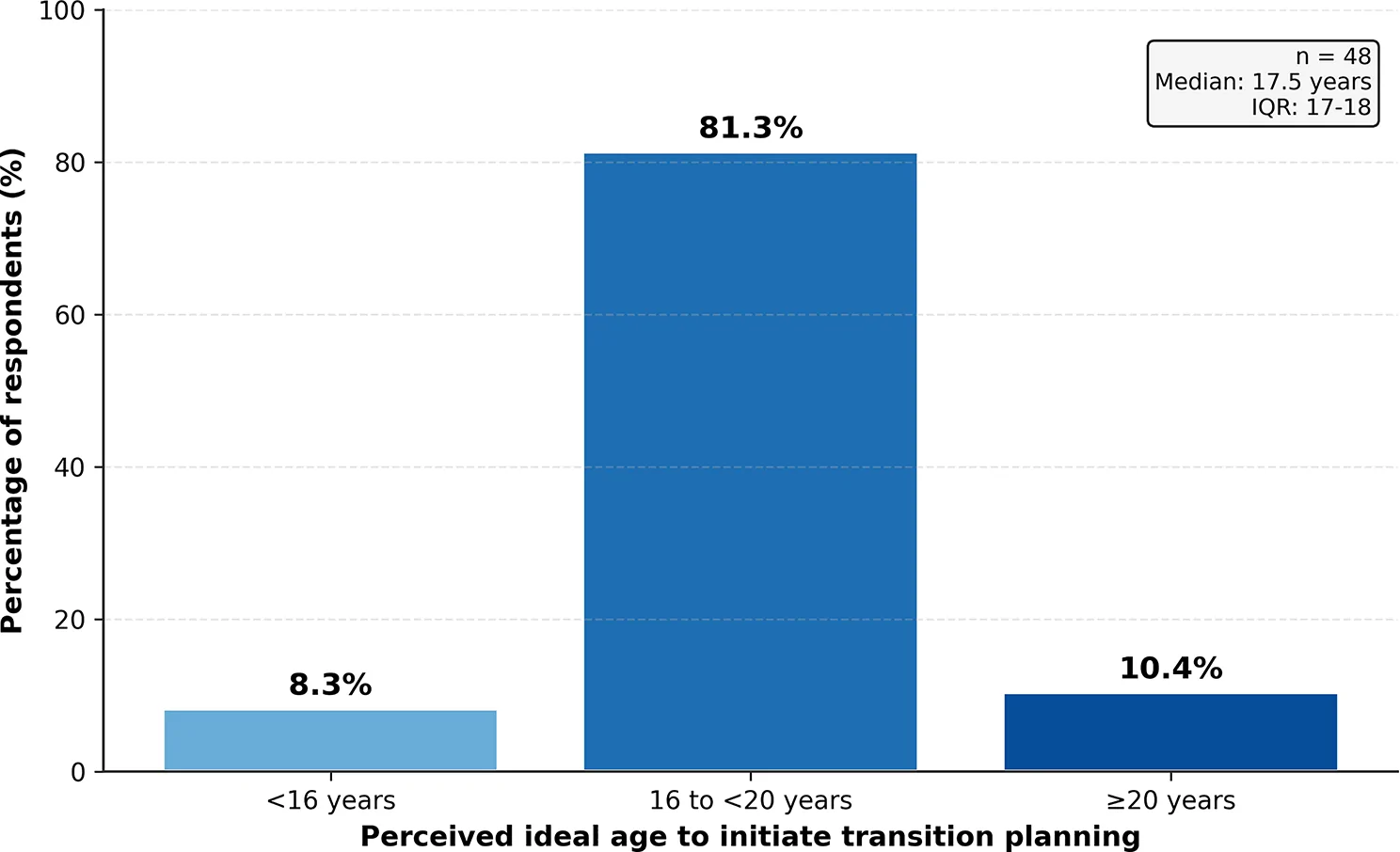
Perceived timing for initiating transition planning in cystinosis

Respondents generally favored initiating transition at later ages. The median recommended starting age was 17.5 years (IQR 17–18; range, 12–22.5 years). Only 4 respondents (8.3%) recommended initiating transition before age 16, 39 (81.3%) placed the ideal starting age between 16 and < 20 years, and 5 (10.4%) proposed starting at 20 years of age or later (Fig. 3).

### Perceived importance and feasibility of specific transition components

**Fig. 4:**
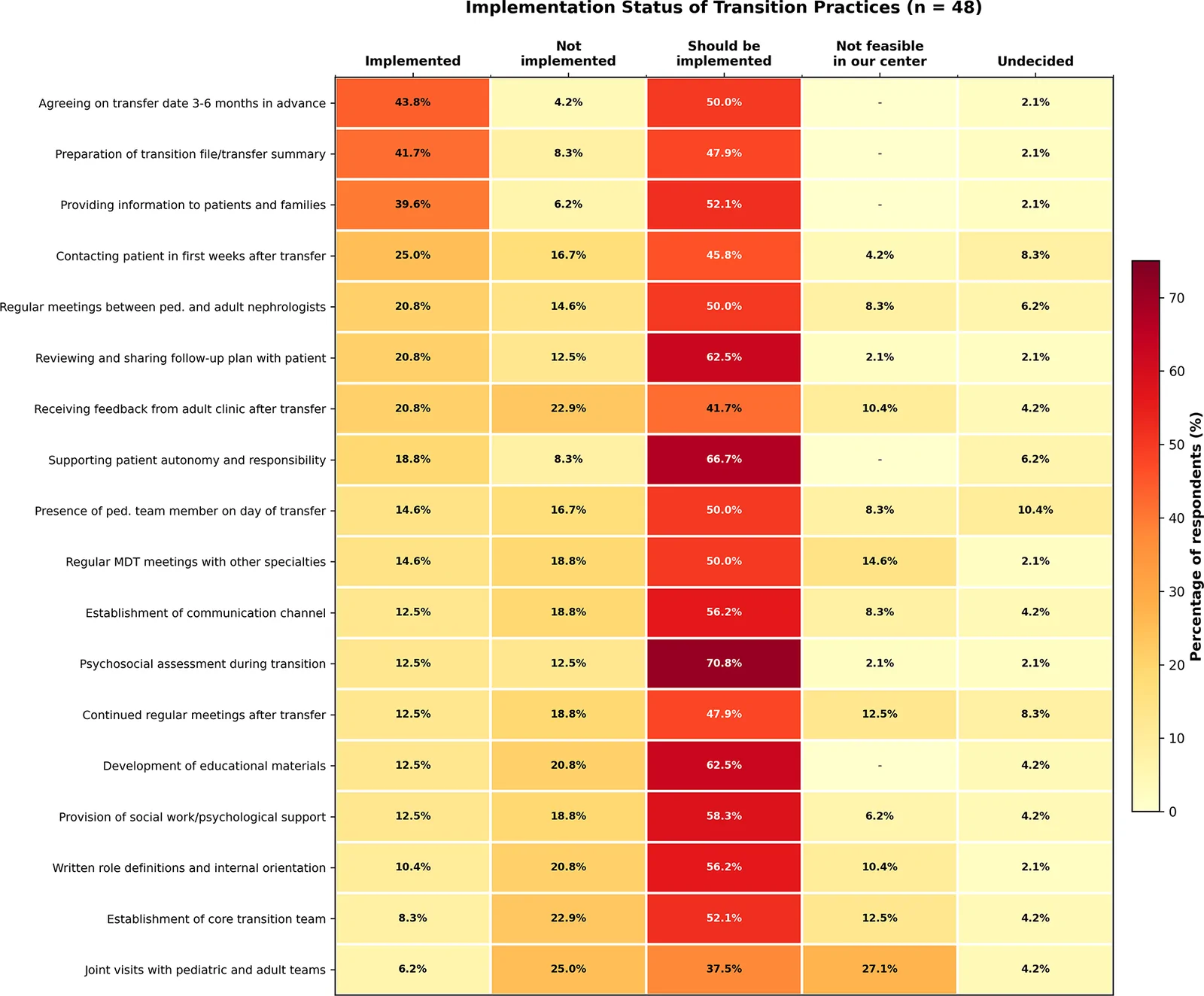
Perceived importance and current implementation of key transition practices as reported by pediatric nephrologists (*n* = 48). Response categories: “Implemented” (currently applied in the respondent’s center), “Not implemented” (not currently applied, without a stated position), “Should be implemented” (not applied but considered necessary), “Not feasible in our center,” and “Undecided/no opinion.” Practices are ranked by implementation rate in descending order. Color intensity reflects percentage values within each cell. A dash (–) indicates 0%

Respondents rated 18 potential components of a cystinosis transition process according to their current practice and perceived feasibility (Fig. 4). The most frequently implemented practices were agreeing on the transfer date 3–6 months in advance (43.8%), preparing a transition file or detailed transfer summary (41.7%), and providing information to patients and families (39.6%). Psychosocial assessment during transition (70.8%) and supporting patient autonomy (66.7%) were the components most frequently endorsed as “should be implemented.” In contrast, joint visits with pediatric and adult teams had the lowest implementation rate (6.2%) and were most frequently deemed not feasible (27.1%).

### Attitudes toward a national structured transition model and willingness to engage

Most respondents supported the development of a national structured transition model for cystinosis in Türkiye. Twenty-eight pediatric nephrologists (58.3%) stated that such a model is definitely necessary, and 16 (33.3%) considered it essential but challenging to implement without external support. The remaining 4 respondents (8.4 %) either preferred a center-specific approach or had no opinion.

**Fig. 5:**
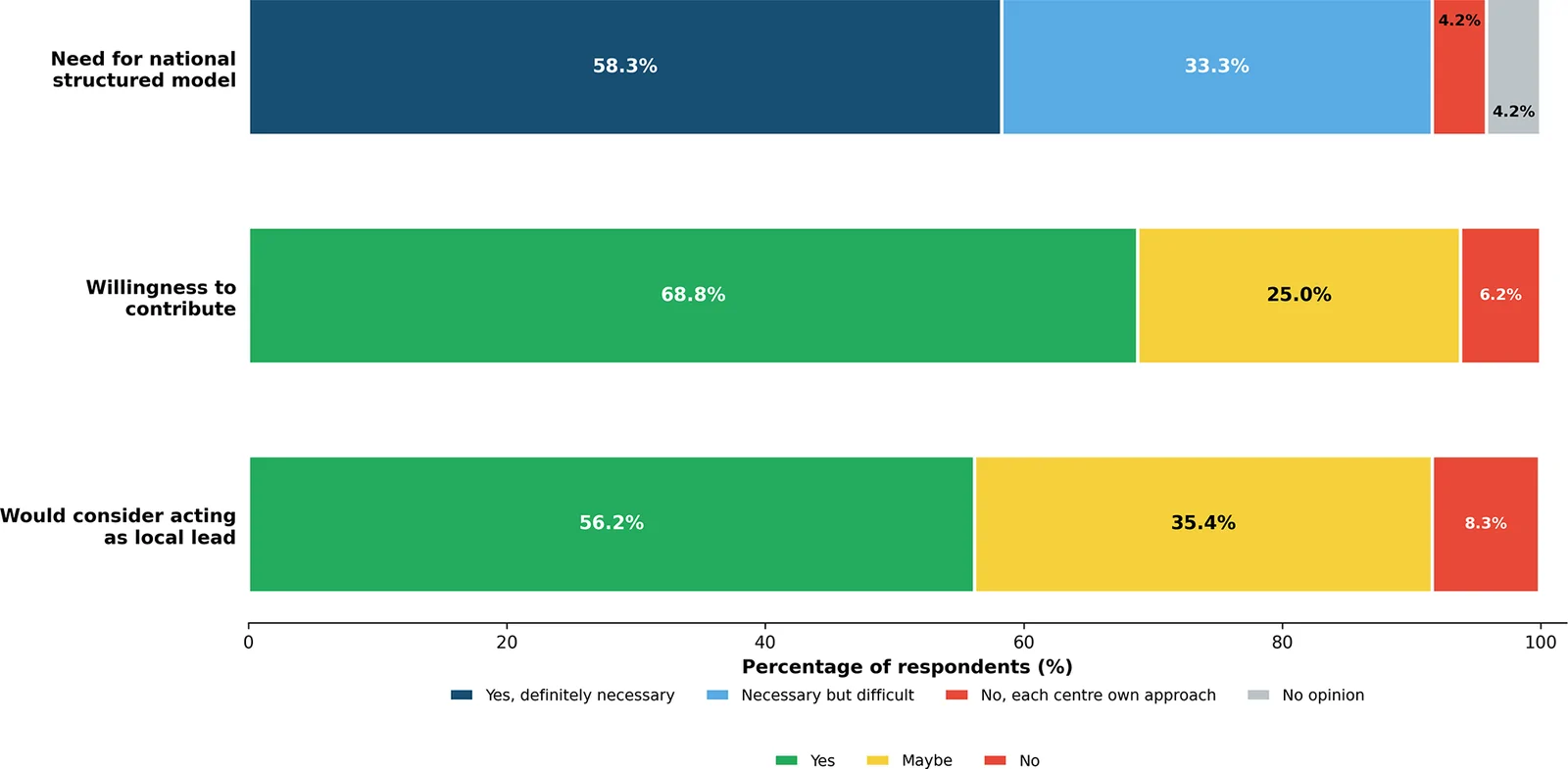
Attitudes toward a national structured transition model, willingness to contribute to the development of a model, and willingness to consider acting as a local lead (*n* = 48). For the first question (need for a national model), response categories were: yes, definitely necessary; necessary but difficult without external support; no, each center should use its own approach; and no opinion (dark blue, light blue, red, and gray, respectively; upper legend). For the second and third questions (willingness to contribute and consideration of acting as a local lead), response categories were: yes, maybe, and no (green, yellow, and red, respectively; lower legend). Percentages represent the proportion of total respondents

Regarding willingness to contribute to the development of a national model, 33 respondents (68.8%) indicated that they would participate, 12 (25.0%) reported that they might contribute, and 3 (6.2%) declined. Of the 48 respondents, 27 (56.2%) indicated they would consider acting as a local lead within their own institution, 17 (35.4%) were uncertain, and 4 (8.3%) declined (Fig. 5).

In exploratory subgroup comparisons, awareness and practice items did not differ significantly by self-rated experience or by current follow-up of an adult-aged patient with cystinosis (all Fisher exact *p* > 0.5; Supplementary Table S1). For example, distinguishing transition from transfer was reported by 12 of 16 (75.0%) very experienced respondents versus 20 of 32 (62.5%) moderately or minimally experienced respondents (*p* = 0.521), and having ever applied a structured transition model by 3 of 16 (18.8%) versus 5 of 32 (15.6%) (*p* = 1.000). Support for a national transition model was high across all subgroups (≥ 86.7%). These patterns suggest that the identified gaps were consistent across experience levels and clinical exposure rather than confined to less experienced clinicians, although the modest sample limits subgroup inference.

## Discussion

This national survey identifies a gap between conceptual awareness of healthcare transition and its routine implementation in cystinosis care. While many pediatric nephrologists framed transition as a planned, longitudinal process rather than a single transfer event, structured and disease-specific transition practices were uncommon. This gap matters because respondents consistently perceived the pediatric-adult interface as a high-risk period, particularly for treatment non-adherence and disruption of multidisciplinary follow-up. Strong support for a national structured transition model, together with a high reported willingness to contribute, suggests that implementation may currently be limited more by organizational and system-level barriers than by lack of clinician engagement.

These findings are consistent with international surveys showing that structured transition care remains incompletely implemented even in well-resourced nephrology systems, indicating that the gaps we observed reflect a broadly shared implementation problem rather than a purely local shortcoming. In a recent national survey of pediatric and adult nephrology programs in the United States, only 20% reported an established transition clinic and 32% had neither a transition clinic nor a formal transition protocol, with resource and financial constraints cited as the dominant barrier and roughly half of paired pediatric–adult programs showing discordant practices [21]. Comparable patterns have been reported from Europe: two years after publication of the ISN/IPNA consensus statement, only about one-third of surveyed pediatric nephrology units had incorporated the guidance into routine practice, most initiated transition planning after 15 years of age, and fewer than half operated a dedicated transition or transfer clinic [22]. A multinational European survey of pediatric kidney transplant centers likewise concluded that the 2011 guidelines were insufficiently implemented, with most patients transferred between 16 and 21 years of age, only about half of centers running a specialized transition clinic, and guideline adherence correlating with national income [23]. Notably, in several of these settings transfer is governed by age-based institutional or national rules; this structural factor, together with pediatric care commonly extending to around 18 years, may partly explain why transition activities tend to be concentrated in late adolescence, consistent with the late median starting age (17.5 years) recommended by our respondents, rather than begun earlier [22, 23].

Viewed against this background, the low frequency of written transition plans (8.3%) and structured model use (16.7%) in our survey are not anomalous but extend a consistent international pattern to the specific context of nephropathic cystinosis and the Turkish health system. Two features nonetheless distinguish our study. Whereas previous surveys addressed transition in chronic kidney disease or kidney transplantation broadly, ours is, to our knowledge, the first to examine clinician awareness and practice specifically for nephropathic cystinosis, a disease whose lifelong multisystem burden and multidisciplinary demands make continuity across the pediatric–adult interface particularly consequential [11, 15]. That clinician engagement in our sample was high while structured practices remained scarce mirrors the resource- and coordination-related constraints emphasized in these international reports [21, 23], locating the principal obstacles to improvement in health-system organization rather than in individual attitudes.

Several features of cystinosis itself compound these system-level challenges. The relevance of transition in cystinosis has increased as the disease has evolved from a historically fatal childhood disorder to a chronic, lifelong multisystem condition [2]. Improvements in cystine-depleting therapy and kidney replacement therapy have enabled increasing numbers of patients to reach adulthood [2, 4–8], shifting the focus of long-term care toward sustained adherence and coordinated monitoring across multiple specialties [15, 18, 19]. Beyond nephrology, comprehensive cystinosis care requires input from ophthalmology, endocrinology, nutrition, and psychosocial services, reflecting the progressive extrarenal burden of the disease [2, 15, 16, 24–26]. Adolescence and emerging adulthood bring their own pressures, including shifting autonomy, educational demands, and the need to navigate healthcare independently. These developmental factors can destabilize medication routines and continuity of surveillance. Qualitative work in cystinosis has described this period as requiring “connected autonomy,” balancing independence with sustained family and professional support [20]. The complex treatment regimen, including pill burden and, in many cases, kidney transplantation, further elevates the risk of non-adherence during this period [18, 27, 28]. Taken together, these challenges argue for structured, disease-specific transition pathways rather than ad hoc transfer [11, 13, 18, 19].

Our survey suggests that multidisciplinary care for pediatric patients with cystinosis in Türkiye is present but unevenly embedded across specialties. Ophthalmology and metabolic medicine were routinely integrated in most centers, whereas psychosocial support services, psychiatry/psychology, and rehabilitation were predominantly accessed on a consultative basis and were reported as unavailable by up to 23% of respondents. Psychosocial and rehabilitative support can shape autonomy, coping, adherence, and social participation, all of which are closely linked to transition readiness [12, 13, 20]. The limited routine access observed in our survey may therefore have direct implications for transition preparedness. Psychosocial transition guidance for cystinosis specifically highlights that high psychological stress and shifting care responsibilities during adolescence complicate treatment adherence and surveillance, further supporting the integration of these services before transfer [12]. If such services are not routinely established during pediatric follow-up, patients may enter adult care with fewer self-management skills and less structured multidisciplinary support, leaving them less prepared for the shift in care models and support structures that accompanies adult services [12, 13].

Our findings also point to limited tracking of continuity across the pediatric–adult interface. Despite substantial pediatric experience in cystinosis care, more than one-third of respondents were unsure whether adult patients with cystinosis were currently followed in their own centers. This uncertainty may reflect genuine discontinuity of information across the pediatric-adult interface, although we can not exclude that it partly stems from the way the question was framed. Together with the low frequency of written transition plans, this suggests that transfers may frequently occur as discrete events rather than as part of a longitudinal shared-care process. Practically, pediatric teams may complete the handover but lack structured feedback loops to confirm ongoing adult follow-up. This matters in cystinosis because sustained multisystem monitoring and adherence support remain necessary well beyond adolescence [4, 18, 27–29]. We did not measure patient-level outcomes or confirm loss to follow-up; however, the combination of unknown adult follow-up status and limited structured transition practices is consistent with possible fragmentation of continuity across services [13, 15, 19].

Another notable finding is the perceived timing of transition. Cystinosis-focused models and renal transition guidance consistently emphasize early, progressive preparation rather than concentrating transition activities around the age of transfer [12, 13, 19]. The RISE protocol recommends structured preparation beginning in adolescence (from approximately 14 years of age), with staged self-management training and communication between pediatric and adult teams [13], while the T-CiS.bcn coordinated model outlines a step-by-step chronogram across adolescence and young adulthood [19]. Psychosocial transition guidance similarly recommends that preparation begin early and progress gradually, noting that programs such as Ready-Steady-Go use staged questionnaires from age 11 onwards [12, 30]. In our survey, however, respondents most often placed the ideal timing for initiating transition in late adolescence, with relatively few selecting ages below 16 years. This pattern suggests that transition may still be conceptualized as a short pre-transfer period rather than a longitudinal readiness-building process. As noted above, structural transfer rules may reinforce this tendency. Starting earlier gives clinicians more time to reinforce adherence routines, address psychosocial barriers, and build structured communication channels between teams before the actual transfer [12, 13, 31].

Respondents endorsed several low-to-moderate burden elements of structured transition. Agreeing on the transfer date in advance, preparing a transition file, and providing information to patients and families were the most frequently implemented practices, while psychosocial assessment and support for patient autonomy were the components most widely endorsed for future implementation. These elements align with renal transition guidance and cystinosis-specific recommendations [12, 13, 15, 18, 19]. In contrast, practices requiring intensive pediatric–adult co-delivery, such as joint visits, had the lowest implementation rate and were most frequently deemed not feasible. Resource and coordination constraints can limit such complex interventions, and transition pathways may be difficult to sustain without dedicated support [13, 19, 32–34]. One pragmatic step would be to prioritize a scalable “minimum bundle” that can be implemented across centers with variable resources. Such a bundle could include (i) a written transition checklist with age-based milestones, (ii) a standardized cystinosis transition/transfer summary template, (iii) a defined communication channel and post-transfer feedback mechanism between pediatric and adult services, and (iv) minimum recommendations for multidisciplinary assessments, including psychosocial screening. Adapting existing cystinosis-focused models to local workflows may facilitate implementation without requiring resource-intensive structures. Although these data derive from a single country, the questions they address are internationally relevant. The pragmatic ‘minimum bundle’ that most respondents considered feasible (a written transition plan, a structured transfer summary, and defined pediatric–adult communication) offers a scalable starting point that could be adapted across settings and, more broadly, to other rare pediatric-onset conditions in which patients increasingly survive into adulthood with a high treatment burden.

This study has both strengths and limitations. It represents the first national survey examining transition practices in cystinosis in Türkiye, and the survey domains were mapped to established transition concepts, renal transition guidance, and cystinosis-specific recommendations, supporting a comprehensive needs assessment. The high proportion of respondents from tertiary academic centers provides representation of settings likely to manage a substantial proportion of patients with cystinosis. However, the sample size was modest, participation was voluntary, and selection bias is possible. The exact response rate could not be calculated due to uncertainty regarding the total number of pediatric nephrologists actively managing cystinosis patients in Türkiye. Responses were self-reported and may be subject to recall and social desirability bias. The questionnaire was developed following a focused literature review and pretested for item clarity but was not formally psychometrically validated. Self-rated experience is subjective and was therefore reported alongside other descriptors such as years of practice and prior clinical experience with cystinosis. We did not include adult nephrology perspectives or patient and family perspectives, which are essential for designing patient-centered and feasible transition pathways. Finally, we did not assess patient-level outcomes; therefore, the findings should be interpreted as evidence of perceived needs, barriers, and practice patterns rather than direct evidence of clinical impact. Findings from the Turkish healthcare context may not be directly generalizable to healthcare systems with different organizational structures. Future studies should incorporate perspectives from adult nephrologists and from patients and families with lived experience of the transition process. Longitudinal data tracking post-transfer follow-up, adherence, and clinical endpoints would help quantify the impact of structured transition approaches. Pilot testing of the proposed “minimum bundle” across centers with varying resources would be a practical next step.

In conclusion, pediatric nephrologists in Türkiye recognize transition in cystinosis as a vulnerable period with high perceived risks of non-adherence and fragmentation of multidisciplinary follow-up. However, structured and disease-specific transition pathways remain uncommon, continuity across the pediatric–adult interface appears insufficiently tracked, and the recommended timing for initiating transition planning tends to be late relative to current guidance. Strong support for a nationally structured transition model and willingness to contribute provide a clear opportunity for action. A feasible, scalable framework, beginning with a standardized minimum set of transition practices adaptable to local resources, may help strengthen continuity and coordination across services in this rare multisystem disease.

## Supplementary Information

Below is the link to the electronic supplementary material.

Supplementary Material 1

## Data Availability

No datasets were generated or analysed during the current study.
