# Supplementary material for: Awareness, perceptions, and practices regarding transition to adult care for nephropathic cystinosis in Türkiye: a nationwide cross-sectional survey of pediatric nephrologists
Source: Orphanet J Rare Dis. 2026 Aug 26;21:358. doi: 10.1186/s13023-026-04557-2 (PMC13673631; doi:10.1186/s13023-026-04557-2)
Supplement: Supplementary file 1 [file 13023_2026_4557_MOESM1_ESM.docx]

**SUPPLEMENTARY MATERIAL 1. Survey questionnaire (English version)**

**Title**

Transition of Care in Patients with Nephropathic Cystinosis in Turkey: Awareness and Clinical Practice

**Purpose**

This survey was designed to assess the level of knowledge, current clinical practices, and perspectives of pediatric nephrologists in Turkey regarding the transition of patients with nephropathic cystinosis from pediatric to adult care as well as their views on the need for a structured transition model.

**Estimated completion time**

5–7 minutes

**Voluntary participation**

Participation is entirely voluntary. You may withdraw from the survey at any time.

**Confidentiality**

No personal identifiers (such as names, institutional names, or contact details) were requested. All responses were used solely for scientific purposes.

**SECTION 1. Respondent and Center Characteristics**

1. Years of professional experience in pediatric nephrology (full years)(Short answer)
2. Type of institution (Single choice)

☐ University hospital

☐ Training and research hospital

☐ State hospital

☐ Private hospital

☐ Other: ____________

1. Number of pediatric nephrologists working in your centre(Short answer) ____ persons
2. Number of adult nephrologists working in your institution (Short answer): ____
3. How experienced do you consider yourself during the follow-up of patients with cystinosis? (Single choice)☐ Very experienced ☐ Moderately experienced☐ Moderately experienced☐ Limited experience☐ No experience.
4. Are there adult patients with cystinosis currently followed up in your center? (Single choice)☐ Yes☐ No☐ Not sure
5. Please indicate how the following specialties are involved in the follow-up of patients with cystinosis at our institution. (Matrix)

**Response options (one per row):**

☐ Routinely follows

☐ Referred when needed

☐ Reviewed once per year

☐ Not followed / Not available

**Specialties:**

- Metabolic medicine
- Endocrinology
- Neurology
- Ophthalmology
- Gastroenterology
- Chest diseases / Pulmonology
- Cardiology
- Urology
- Physical therapy / Rehabilitation
- Dietetics
- Psychiatry / Psychology
- Social work

**SECTION 2. Knowledge and Awareness**

1. Do you think there is a difference between the concepts of “transition” and “transfer”? (Single choice)

☐ Yes

☐ No

☐ Not sure

2. Have you previously heard the term “structured transition process”? (Single choice)

☐ Yes

☐ No

3. Do you have any knowledge of a model, guideline, or program related to the transition in patients with cystinosis? (Single-choice)

☐ Yes, I have detailed knowledge

☐ I have limited knowledge

☐ I have no knowledge

4. Are you aware of the risks associated with the transitional process in patients with cystinosis? (Single-choice)

☐ Yes, in detail

☐ In general terms

☐ No

☐ I have never considered this

1. Do you consider the following challenges encountered by patients with cystinosis during the transition process? (Matrix)Response options (one per row)

☐ Yes

☐ No

☐ Not sure / Have not considered

Items:

- Disruption of multidisciplinary follow-up
- Non-adherence to treatment (medication or missed appointments)
- Psychosocial stress (identity issues, independence, etc.)
- Social security or insurance-related problems
- Difficulties adapting to education and employment

1. Do you think there are systemic differences between pediatric and adult clinics? (Single-choice)

☐ Yes

☐ No

☐ I have never considered this

5. Do you think there are differences between pediatric and adult clinics in the following areas? (Matrix)Response options (one per row):☐ Marked difference☐ Limited difference☐ No difference / Not sure

**Domains:**

- Follow-up model
- Frequency of visits
- Multidisciplinary structure
- Treatment support
- Communication style

**SECTION 3. Current Clinical Practice**

1. Have you or your team ever implemented a structured transition model for patients with cystinosis? (Single-choice)

☐ Yes

☐ No

☐ Planning to do so

☐ Not sure / No opinion

2. How is the transition process for patients with cystinosis currently organized in your center? (Single-choice)

☐ A structured and written transition plan is implemented

☐ A partially structured process is in place

☐ Transfer is based solely on a discharge summary

☐ No transition-specific practice is applied

3. In your opinion, at what age or age range should the transition process ideally be initiated in patients with cystinosis?(Short answer) ________ (e.g. 6–10; 15–18; 18–20)
4. Do you consider the following practices important for the transition process of patients with cystinosis?(Regardless of whether they are currently applied at your institution). (Matrix)Response options (one per row)

☐ Implemented

☐ Not implemented

☐ Should be implemented

☐ Not feasible in our center

☐ Undecided / No opinion

**Practices:**

- Establishment of a core transition team
- Written role definitions and internal orientation
- Establishment of a fixed communication channel (e-mail, phone chain, etc)
- Regular meetings or boards between paediatric and adult nephrologists
- Joint visits with paediatric and adult teams
- Regular (at least annual) multidisciplinary meetings with other specialties
- Providing information to patients and families about the transition process
- Psychosocial assessment during transition
- Supporting patient autonomy and responsibility
- Development of age and developmentally appropriate educational materials
- Reviewing and sharing the follow-up plan with the patient
- Provision of social work or psychological support
- Preparation of a transition file or detailed transfer summary
- Agreeing on the transfer date at least 3–6 months in advance
- Presence of a paediatric team member on the day of transfer
- Contacting the patient in the first weeks after transfer
- Receiving feedback from the adult clinic after transfer
- Continued regular meetings between paediatric and adult teams after transfer

**SECTION 4. Opinions and Suggestions**

1. Do you think that a nationally structured transition model for cystinosis is needed in Turkey? (Single-choice)

☐ Yes, definitely needed

☐ Needed but difficult to implement without external support

☐ No, each center should use its own approach

☐ No opinion

2. Would you be willing to contribute to developing this model? (Single-choice)

☐ Yes

☐ Maybe

☐ No

3. Would you consider acting as a local lead for such a model in your institution? (Single-choice)

☐ Yes

☐ Maybe

☐ No

**Optional contact (not linked to survey responses)**

Participants may share additional comments or suggestions via e-mails. Messages sent by e-mail were not linked to survey responses and were not included in the analytical dataset.

Thank you for your participation.

**Supplementary Table S1.** **Exploratory subgroup comparisons of key awareness and practice items (N = 48).**

**A. By self-rated cystinosis experience**

| **Item** | **Very experienced (n = 16)** | **Moderately / minimally experienced (n = 32)** | **p*** |
| --- | --- | --- | --- |
| Distinguishes transition from transfer | 12 (75.0) | 20 (62.5) | 0.52 |
| Heard of “structured transition process” | 9 (56.2) | 15 (46.9) | 0.76 |
| Any knowledge of cystinosis transition models | 8 (50.0) | 15 (46.9) | 1.000 |
| Ever applied a structured transition model | 3 (18.8) | 5 (15.6) | 1.000 |
| Supports a national transition model | 14 (87.5) | 30 (93.8) | 0.59 |

**B. By current follow-up of an adult-aged patient with cystinosis**

| **Item** | **Yes (n = 15)** | **No / unsure (n = 33)** | **p*** |
| --- | --- | --- | --- |
| Distinguishes transition from transfer | 11 (73.3) | 21 (63.6) | 0.74 |
| Heard of “structured transition process” | 7 (46.7) | 17 (51.5) | 1.000 |
| Any knowledge of cystinosis transition models | 8 (53.3) | 15 (45.5) | 0.75 |
| Ever applied a structured transition model | 3 (20.0) | 5 (15.2) | 0.69 |
| Supports a national transition model | 13 (86.7) | 31 (93.9) | 0.57 |

*Values are n (%). *p values from Fisher exact tests. All comparisons are exploratory and interpreted descriptively given the limited sample size; no comparison reached statistical significance. The number of cystinosis patients followed per clinician was not collected, so patient volume could not be examined directly; self-rated experience and current follow-up of an adult-aged patient with cystinosis were used as available proxies for experience and transition-related exposure.*
